# SESN2 protects against denervated muscle atrophy through unfolded protein response and mitophagy

**DOI:** 10.1038/s41419-021-04094-9

**Published:** 2021-08-24

**Authors:** Xiaofan Yang, Pingping Xue, Meng Yuan, Xiang Xu, Cheng Wang, Wenqing Li, Hans-Günther Machens, Zhenbing Chen

**Affiliations:** 1 Department of Hand Surgery, Union Hospital, Tongji Medical College, Huazhong University of Science and Technology, Wuhan, 430022 China; 2 Department of Pharmacy, Tongji Hospital, Tongji Medical College, Huazhong University of Science and Technology, Wuhan, 430030 China; 3 Department of Hand and Foot Surgery, Huazhong University of Science and Technology Union Shenzhen Hospital, Shenzhen, Guangdong China; 4 Department of Plastic and Hand Surgery, Technical University of Munich, Munich, 81675 Germany

**Keywords:** Neurological disorders, Trauma

## Abstract

Denervation of skeletal muscles results in a rapid and programmed loss of muscle size and performance, termed muscle atrophy, which leads to a poor prognosis of clinical nerve repair. Previous researches considered this process a result of multiple factors, such as protein homeostasis disorder, mitochondrial dysfunction, endoplasmic reticulum stress (ERS), and apoptosis, while their intrinsic association remains to be explored. In this study, Sestrin2 (SESN2), a stress-inducible protein, was shown to act as a key protective signal involved in the crosstalk therein. SESN2 expression was induced in the gastrocnemius two weeks post denervation, which was accompanied by ERS, mitochondrial dysfunction, and apoptosis. Knockdown of SESN2 aggravated this situation and resulted in severer atrophy. Similar results were also found in rotenone-treated C2C12 cells. Furthermore, SESN2 was demonstrated to be induced by an ERS-activated transcription factor CCAAT-enhancer-binding protein beta (C/EBPβ). Once induced, SESN2 halted protein synthesis by inhibiting the mammalian target of rapamycin complex 1 (mTORC1), thereby attenuating ERS. Moreover, increased SESN2 activated the specific autophagic machinery and facilitated the aggregation of sequestosome 1 (SQSTM1, p62) on the mitochondrial surface, which promoted the clearance of damaged mitochondria through mitophagy. Collectively, the SESN2-mediated unfolded protein response (UPR) and mitophagy play a critical role in protecting against denervated muscle atrophy, which may provide novel insights into the mechanism of skeletal muscle atrophy following denervation.

## Introduction

Peripheral nerve injury caused by trauma accounts for 2% of all limb injuries [1]. With the development of microsurgical technology, nerve anastomosis and transplantation have become the main methods to restore the anatomical structure of peripheral nerve, however, one-third of all peripheral nerve injuries achieve poor functional recovery [2]. Muscle atrophy, which is characterized by progressive reduction of muscle fiber cross-sectional area and protein content, develops rapidly with profound weakness after denervation and is responsible for the poor prognosis of nerve repair [3]. Prior investigations considered it a result of protein homeostasis disorder, which exhibited decreased protein synthesis together with enhanced protein degradation through ubiquitin–proteasome system (UPS) as well as autophagy, while the molecular mechanisms governing this balance remained to be explored [4]. Recent studies attributed this process to the dysfunction of specific organelles like endoplasmic reticulum (ER, also called sarcoplasmic reticulum) and mitochondria [5, 6]. However, how these events occurred and affected muscle atrophy remained unclear.

Mitochondria play central roles in cell energy supply through oxidative phosphorylation. Other functions of mitochondria include lipid synthesis, signal transduction and calcium homeostasis maintenance [7]. However, mitochondria are also the main source of reactive oxygen species (ROS) and trigger apoptosis by releasing the proapoptotic factor cytochrome c [8]. Mitochondrial autophagy, namely mitophagy, is the main way of mitochondrial quality control. Dysfunctional mitochondria, which are able to generate ROS and induce cell death, will be recognized and removed by autophagy, selectively [9]. In our previous study, mitophagy activation was found in mice gastrocnemius after denervation [10], while its correlations with ERS and muscle atrophy need to be further described.

ER serves an indispensable role in protein folding and calcium homeostasis. However, the accumulation of unfolded and misfolded proteins in ER lumen causes ERS. As a protective response, three transmembrane sensor molecules—protein kinase RNA-like ER kinase (PERK), inositol-requiring enzyme 1 (IRE1), and activating transcription factor 6 (ATF6) are activated to initiate unfolded protein response (UPR) [11], which relieves ERS by suppressing protein synthesis, accelerating protein degradation, and increasing molecular chaperones. However, persistent ERS that exceeds the capacity of UPR may still lead to various pathological consequences including apoptosis [12]. As shown in numerous studies, ERS-induced UPR fulfils vital roles in muscle differentiation, regeneration, and mass control [13, 14]. However, the detailed mechanism mediating the protective effect of UPR on denervated muscle atrophy remains to be explored.

Sestrins are stress-inducible proteins that prevent ROS- or ERS-associated pathologies by suppressing mTORC1 through activation of AMP-activated protein kinase (AMPK) [15]. Among three Sestrin homologs (SESN1–3) in mammals, we recently found that SESN2 expression in skeletal muscle was elevated upon denervation and knockdown of SESN2 aggravated muscle atrophy. Further studies demonstrated that SESN2 was transcriptionally activated through PERK–C/EBPβ pathway upon denervation-associated ERS and in turn maintained ER homeostasis in muscle by suppressing mTORC1-mediated protein synthesis. Besides, SESN2 activated the specific autophagic machinery and facilitated the aggregation of p62 on the mitochondrial surface, which promoted the clearance of damaged mitochondria through mitophagy and therefore relieved the oxidative stress and apoptosis downstream. Our results suggested that SESN2 critically mediated skeletal muscle adaptation to mitochondrial dysfunction and ERS and was the endogenous attenuator of denervated muscle atrophy that operated primarily through maintaining ER homeostasis and mitochondria quality.

## Results

### Denervation led to atrophy of the dominated muscles

**Fig. 1:**
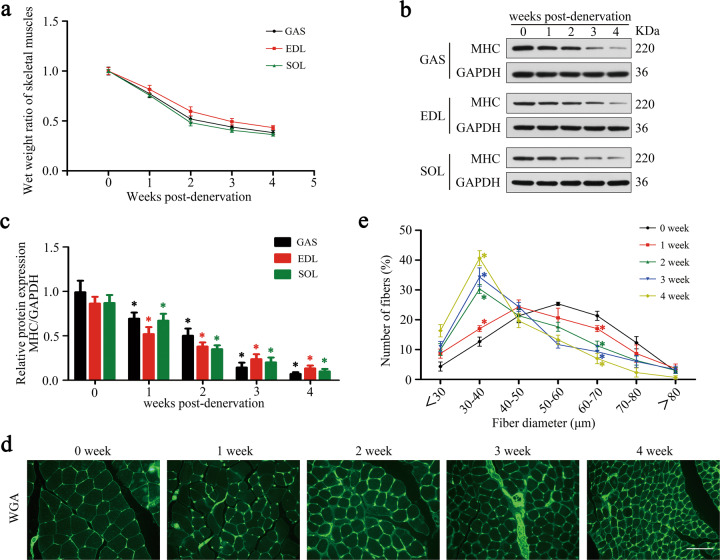
Denervation led to atrophy of the dominated muscles. **a** The wet-weight ratio (the weight of the operational side divided by the contralateral side) of GAS, EDL, and SOL at the indicated time points post denervation. **b, c** Western blot analysis of the dynamic changes of MHC protein expression after denervation. **d, e** Quantification of gastrocnemius fiber diameter by immunofluorescence staining of WGA. Scale bar 50 μm. Data were presented as mean ± SD. *n* = 6. *P < 0.05 vs control (zero week).

To uncover the progress of denervated muscle atrophy, mice sciatic nerve transection model was established and different types of skeletal muscles were analyzed therein. As previously reported [16], muscle mass of gastrocnemius (GAS, mixed-type muscle), extensor digitorum longus (EDL, fast-twitch muscle containing type IIB and IID fibers), and soleus (SOL, slow-twitch muscle containing type I and IIA fibers) decreased rapidly upon denervation, during which the atrophic process displayed a typical two-stage feature with a rapid weight loss (about 47%) during the first two weeks and then a milder decrease (about 14%) within the next two weeks (Fig. 1a). As an important structural protein in muscle fiber, myosin heavy chain (MHC) was further detected by western blot and revealed a similar variation as before (Fig. 1b, c). Finally, fiber-diameter analysis was performed by fluorescence staining of wheat germ agglutinin (WGA), the results indicated clearly that denervation drove the fibers to diminish with a gradually lesser diameter (Fig. 1d, e). In general, skeletal muscles, regardless of the muscle types, atrophied rapidly after denervation.

### Protective role of SESN2 against atrophy after denervation

**Fig. 2:**
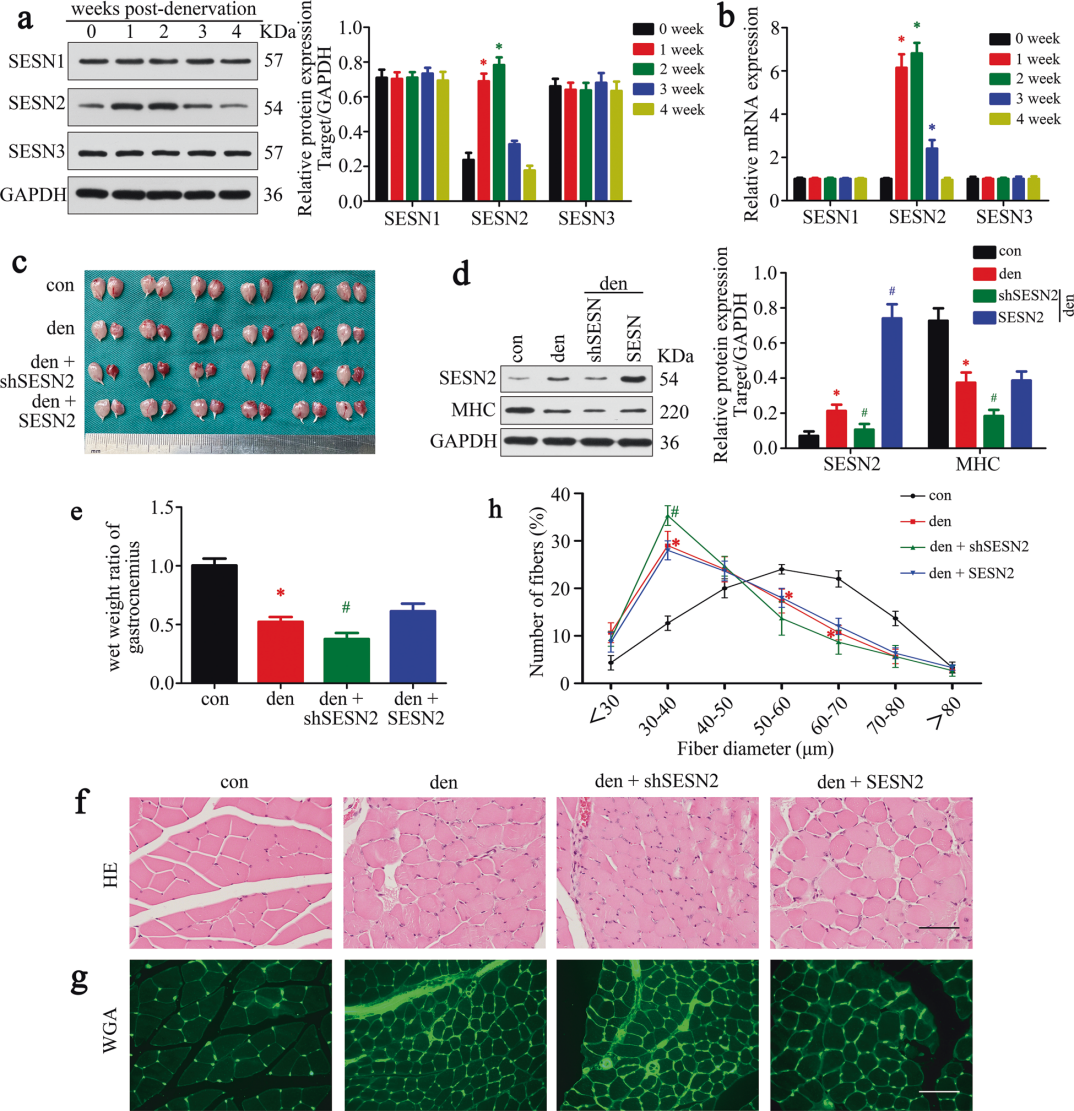
Protective role of SESN2 against atrophy after denervation. **a, b** The contents of SESN1–3 were analyzed by western blot and RT-PCR. **c** GAS of different groups harvested 2 weeks post denervation. **d** Western blot analysis confirmed the expressions of SESN2 and MHC. **e** Wet-weight ratio of GAS in different groups. **f** Morphological observation of GAS muscles in different groups by H&E staining. Scale bar 50 μm. **g, h** Quantification of muscle fiber diameter by immunofluorescence staining of WGA. Scale bar 50 μm. Data were presented as mean ± SD. *n* = 6. *P < 0.05 vs control (zero week or sham-operation group). ^#^*P* < 0.05 vs denervation group. Den, denervation; Con, control.

To clarify the role of Sestrin family in muscle atrophy, three Sestrin homologs in GAS post denervation were detected. Interestingly, prominent accumulation of SESN2 mRNA and protein was observed in the first two weeks post denervation, followed by a gradual reduction in the next two weeks. No obvious variation of SESN1 and SESN3 expression was observed (Fig. 2a, b and Supplementary Table 1). Given that the atrophy of denervated muscles progressed primarily during the first two weeks coincidentally, we inferred that SESN2 might get involved in the process of muscle atrophy.

Next, SESN2 knockdown and overexpression models were generated to investigate the involvement of SESN2 in the atrophy of denervated GAS. GAS samples were collected two weeks post denervation (Fig. 2c). SESN2 expression was confirmed by western blot (Fig. 2d). MHC expression, muscle mass evaluation, and H&E staining revealed that muscle atrophy was significantly aggravated after SESN2 knockdown and was slightly reversed by SESN2 overexpression (with no significance) (Fig. 2d–f). Fiber-size analysis further indicated that the fiber diameter diminished in denervated GAS and was aggravated by AAV-shSESN2 injection (Fig. 2g, h). Together, the above results indicated that SESN2 was induced and played a protective role in GAS against atrophy after denervation.

### Knockdown of SESN2 aggravated ERS, mitochondrial dysfunction, and apoptosis in denervated GAS

**Fig. 3:**
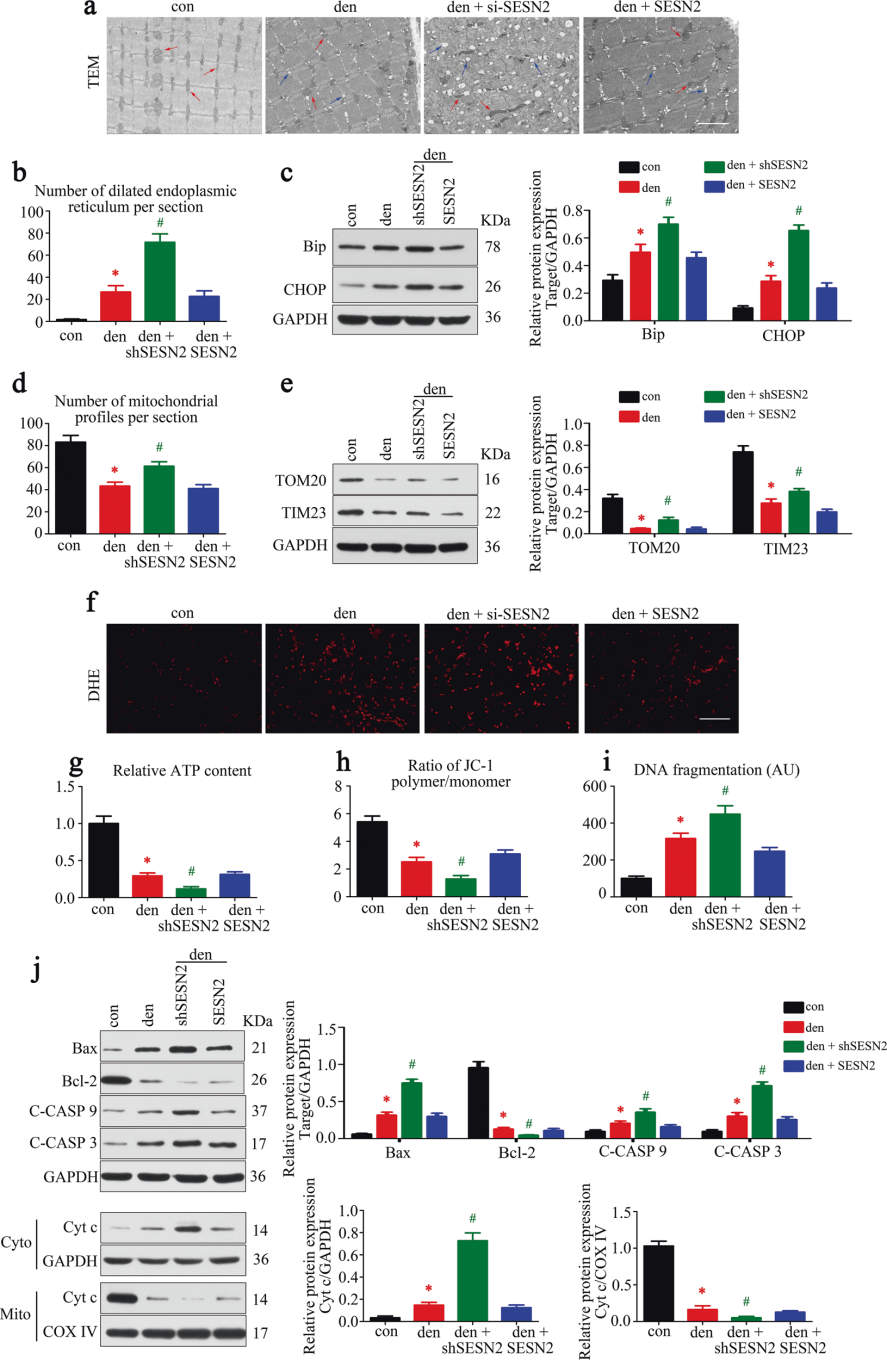
Knockdown of SESN2 aggravated ERS, mitochondrial dysfunction, and apoptosis in denervated GAS. **a** Microstructure of GAS was observed by TEM. Red and blue arrows indicated mitochondria and dilated ER, respectively. Scale bar 2 μm. **b** The number of dilated ER was statistically analyzed. **c** ERS hallmarks—Bip and CHOP were quantified by western blot. **d** The number of mitochondrial profiles in each group were statistically analyzed. **e** Mitochondrial membrane proteins TOM20 and TIM23 were quantified by western blot. **f–h** Mitochondrial function was evaluated comprehensively by ROS, ATP, and Δψm detection. Scale bar 25 μm. **i** Apoptosis in GAS was assessed by detecting DNA fragmentation. **j** Western blot analysis of the protein changes related to mitochondria-dependent apoptosis. Cytochrome c in cytosolic and mitochondrial subfractions was assessed respectively. Data were presented as mean ± SD. *n* = 6. *P < 0.05 vs control group. ^#^*P* < 0.05 vs denervation group. Den denervation, Con control, C-CASP 9 cleaved caspase-9, C-CASP 3 cleaved caspase-3, Cyto cytoplasm, Mito mitochondria; Cyt c cytochrome c.

To further explore the mechanism underlying muscle atrophy after denervation, we focused on the ultrastructural changes of skeletal muscle (Fig. 3a). Obvious dilation of ER was observed in denervated GAS, which was more common after SESN2 inhibition (Fig. 3b). Then we detected the ERS hallmarks—binding immunoglobulin protein (Bip) and C/EBP homologous protein (CHOP) and obtained consistent results (Fig. 3c). Mitochondrial disorder was also evident in denervated GAS. The remarkable decrease of mitochondria number was observed after denervation (Fig. 3d), which was further confirmed by the reduction of mitochondrial outer-/inner-membrane protein TOM20/TIM23 (Fig. 3e). Mitochondrial function was then detected and achieved consistent results, showing the accumulation of ROS, reduction of ATP content and loss of Δψm (Fig. 3f–h). Interestingly, knockdown of SESN2 reversed the decrease of mitochondria number induced by denervation, but further aggravated mitochondrial dysfunction, which might be explained by the accumulation of dysfunctional mitochondria.

As previous research reported that apoptosis was involved in denervated muscle atrophy [17], quantitative analysis of DNA fragmentation by cell death ELISA was performed in GAS. As expected, a significant increase of apoptosis was observed in denervated GAS, and was further aggravated by SESN2 knockdown (Fig. 3i). Given that ERS and mitochondrial dysfunction were both able to induce apoptosis through the mitochondria-dependent pathway [18], western blot was then carried out to analyze activities of the mitochondria-dependent pathway. As shown in Fig. 3j, increase of pro-apoptotic proteins (Bax, cleaved caspase-9, and cleaved caspase-3) and decrease of antiapoptotic protein (Bcl-2) were observed in denervated GAS and further exacerbated by SESN2 knockdown. Furthermore, cytosolic and mitochondrial cytochrome-c content was quantified respectively to evaluate cytochrome-c liberation. Consistently, a characteristic distribution of cytochrome c (higher levels in the cytoplasm and lower levels in mitochondria [19]) was found in denervated GAS and emphasized after SESN2 knockdown. In total, these results indicated that SESN2 was required for ER homeostasis and mitochondrial quality control, and it could also resist the mitochondria-dependent apoptosis in denervated GAS.

### Knockdown of SESN2 aggravated ERS, mitochondrial dysfunction, and apoptosis induced by rotenone in C2C12 cells

**Fig. 4:**
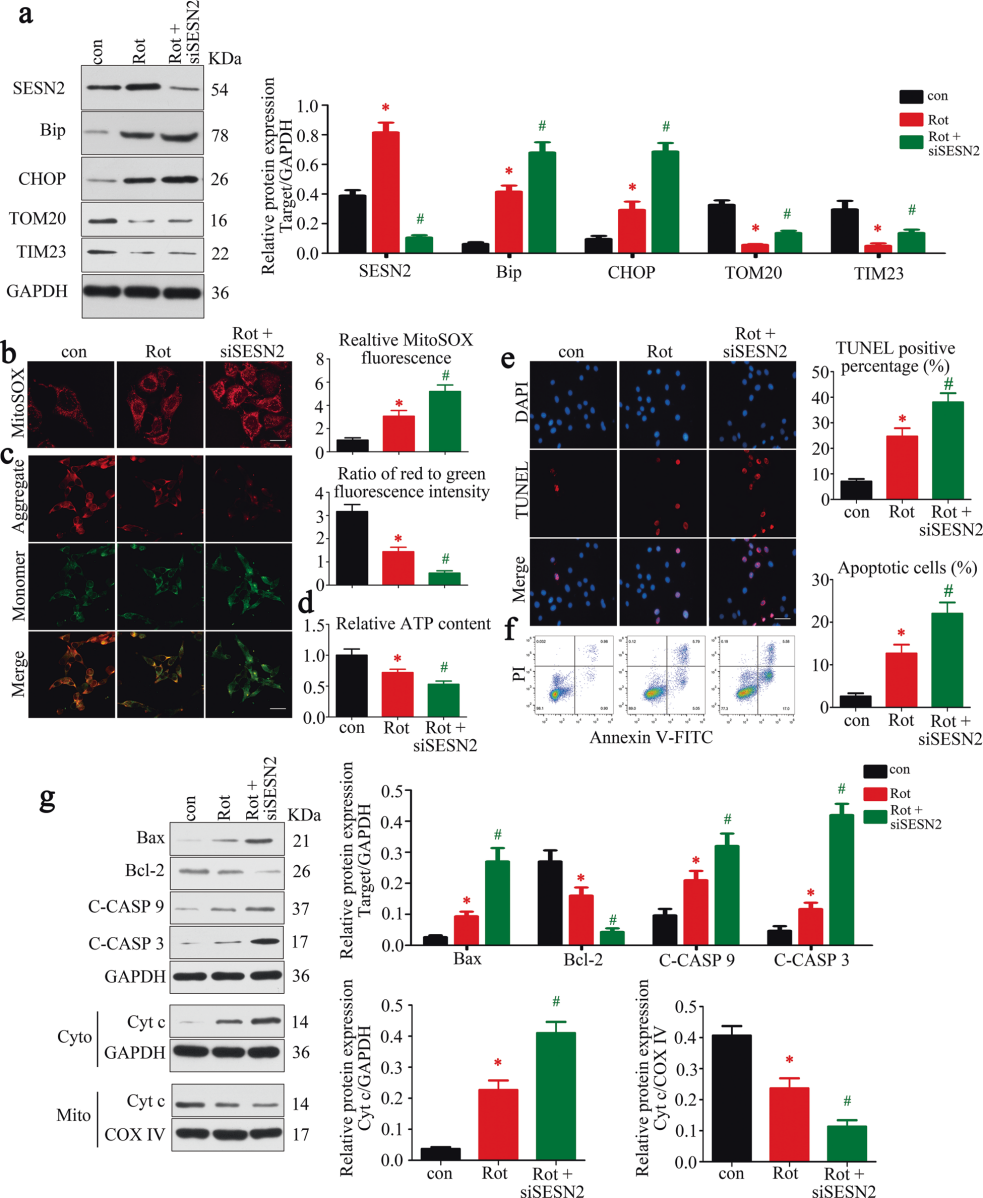
Knockdown of SESN2 aggravated ERS, mitochondrial dysfunction, and apoptosis induced by rotenone in C2C12 cells. **a** Western blot analysis examined the effect of siSESN2. ER and mitochondria homeostasis were investigated by Bip, CHOP, TOM20, and TIM23 separately. **b** MitoSOX Red labeling detected the mtROS levels under different treatment. Scale bar 10 μm. **c** Determination of Δψm using the JC-1 probe. Red and green fluorescence represented the aggregate and monomeric form of JC-1, respectively. The ratio of red-to-green fluorescence intensity was calculated. Scale bar 50 μm. **d** Relative content of ATP. **e, f** TUNEL and FCM assay indicated the apoptosis of C2C12 cells in each group. The apoptotic rate was further analyzed. Scale bar 50 μm. **g** Western blot analysis of the protein changes related to mitochondria- dependent apoptosis. Cytochrome c in cytosolic and mitochondrial subfractions was assessed respectively. Data were presented as mean ± SD. *P < 0.05 vs control (0 nM rotenone treatment). ^#^*P* < 0.05 vs rotenone treatment. Con control, Rot rotenone, C-CASP 9 cleaved caspase-9, C-CASP 3 cleaved caspase-3, Cyto cytoplasm, Mito mitochondria, Cyt c cytochrome c.

To verify the protective role of SESN2 for ERS, mitochondrial dysfunction, and apoptosis in vitro, knockdown of SESN2 was obtained in rotenone-treated C2C12 cells. As expected, ERS, mitochondrial dysfunction, and apoptosis was activated in rotenone-treated C2C12 cells in a concentration-dependent manner, similar results were also achieved in tunicamycin-treated cells (Supplementary Figs. 1 and 2). About 100 nM rotenone (a mitochondrial electron-transport chain complex-I inhibitor) was then adopted in the next experiments. Here we observed a higher degree of ERS in SESN2-knockdown cells by quantitative analysis of Bip and CHOP (Fig. 4a). Furthermore, SESN2 knockdown aggravated the rotenone-induced mitochondrial dysfunction (mtROS overproduction, Δψm depolarization, and ATP reduction) (Fig. 4b–d). These results demonstrated that SESN2 was essential for ER homeostasis and mitochondrial quality control. Next, we tested whether SESN2 was involved in rotenone-induced C2C12 cell apoptosis. From TUNEL and FCM results, C2C12 cell apoptosis was induced by rotenone and significantly accelerated after SESN2 knockdown (Fig. 4e, f). Quantitative analysis of the apoptosis-signaling pathway-associated proteins revealed that the mitochondria-dependent apoptosis was activated in rotenone-treated C2C12 cells and achieved a higher level by siSESN2, similar to the in vivo experiments (Fig. 4g). These data proved that SESN2 was required to prevent C2C12 cell apoptosis induced by rotenone.

### PERK–C/EBPβ axis mediated SESN2 induction upon ERS

**Fig. 5:**
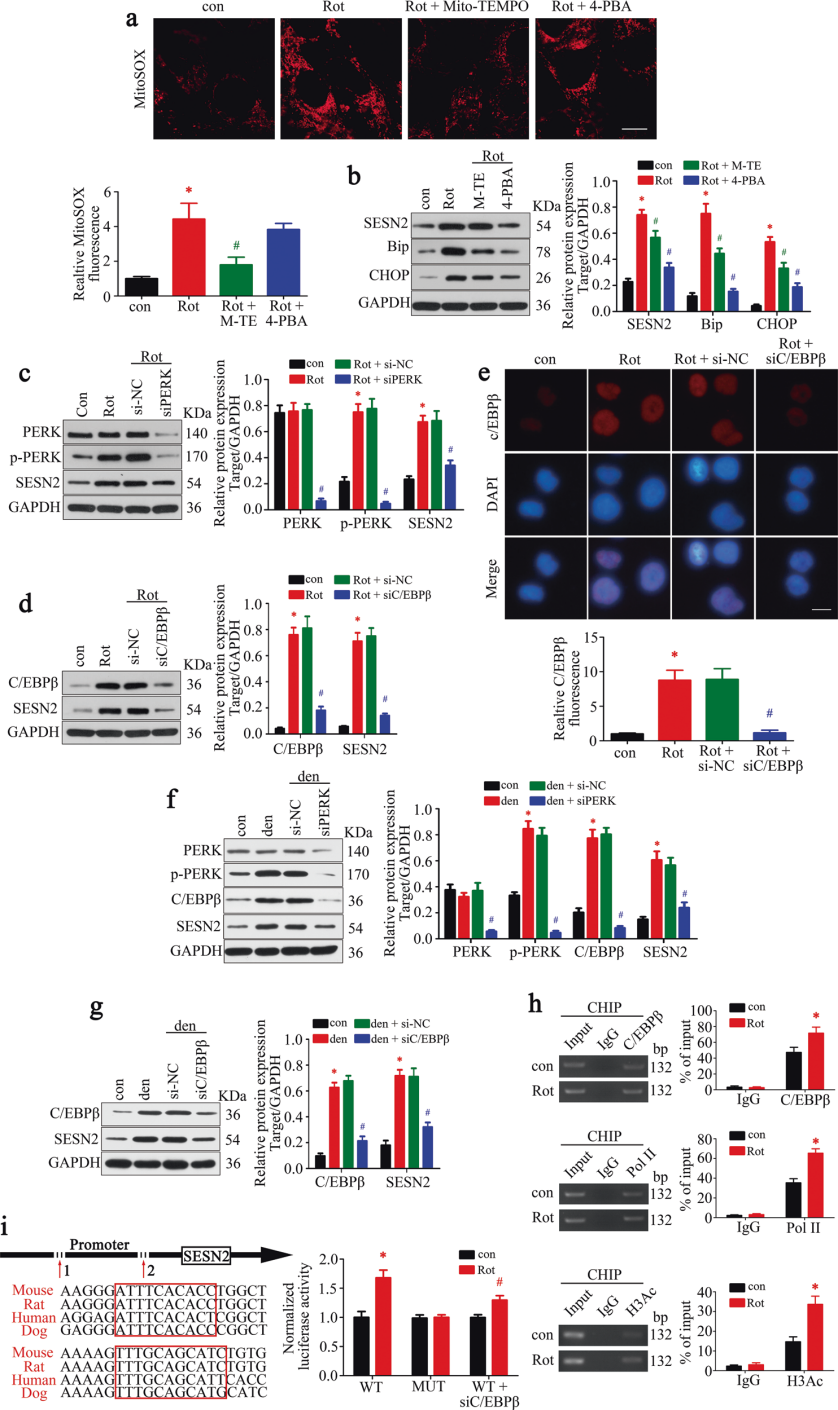
PERK–C/EBPβ axis mediated SESN2 induction upon ERS. **a** MitoSOX Red labeling detected the mtROS levels under treatment of Mito-TEMPO and 4-PBA. Scale bar 10 μm. **b** Western blot analysis of SESN2 and ERS hallmarks—Bip and CHOP. **c, d** Knockdown of PERK and C/EBPβ was achieved and their regulatory effect on SESN2 expression was confirmed by western blot. **e** Nuclear accumulation of C/EBPβ in response to rotenone treatment was detected by immunofluorescence staining. Scale bar 10 μm. **f, g** The effect of PERK–C/EBPβ axis on SESN2 expression was further validated in denervated GAS. **h** ChIP assay with anti-C/EBPβ antibody was performed to verify the binding between C/EBPβ and SESN2 promoter in C2C12 cells under control and rotenone treatment. ChIP with anti-Pol II and anti-acetylated histone H3 antibody was performed as positive controls. **i** Schematic representation of the proximal promoter region of SESN2 gene. The C/EBPβ-binding sites in the SESN2 promoter were marked in red box, and DNA sequences of the sites are shown for four different mammalian species. C2C12 cells were transfected with luciferase reporter containing wild-type (WT) or C/EBPβ-binding site-mutated (MUT) SESN2 promoter DNA sequence. About 48 h after transfection, cells were incubated with rotenone for 24 h and subjected to luciferase assay. Data were presented as mean ± SD. *n* = 6. *P < 0.05 vs control. ^#^*P* < 0.05 vs rotenone treatment or denervation. Con control, Rot rotenone, Den denervation, H3Ac acetylated histone H3.

To understand how SESN2 was induced upon denervation, specific inhibition of mtROS and ERS (known as SESN2 triggers) were performed. As in Fig. 5a, b, Mito-TEMPO (a mitochondria-targeted ROS scavenger) administration significantly suppressed mtROS production and ERS (shown by reduced Bip and CHOP levels) in C2C12 cells. Further, the rotenone-induced SESN2 elevation was attenuated by Mito-TEMPO. 4-phenyl butyric acid (4-PBA, ERS inhibitor) markedly attenuated ERS, but exerted no obvious effect on mtROS production. However, it created a greater decline of SESN2 in C2C12 cells than Mito-TEMPO. These results suggested that ERS was activated by mtROS and was the main upstream trigger of SESN2 in rotenone-treated C2C12 cells.

Next, we wonder how SESN2 was induced by ERS and what the underlying mechanism was. As shown in Fig. 5c, d and Supplementary Fig. 3, knockdown of the ERS sensor PERK, but not IRE1 and ATF6, significantly suppressed the rotenone-induced SESN2 expression, though all of them were elevated after rotenone treatment. Subsequently, the involvement of transcription factors downstream of PERK was further investigated. Interestingly, C/EBPβ, which accumulated inside the nucleus after rotenone treatment (Fig. 5e), was necessary and sufficient to mediate rotenone-induced SESN2 expression. However, whether ATF4 and CHOP were blocked or not makes no difference to SESN2 expression. To further validate the role of PERK-C/EBPβ pathway, knockdown of PERK and C/EBPβ was carried out in denervated GAS and similar results were achieved (Fig. 5f, g). Next, the chromatin-immunoprecipitation (ChIP) assay was performed to verify the binding of C/EBPβ on SESN2 promoter in C2C12 cells. Compared with the sample bounded with IgG, we found that C/EBPβ-bound complex displayed a remarkable enrichment of SESN2 promoter after rotenone treatment. ChIP with anti-Pol II and anti-acetylated histone H3 antibody was performed as positive controls (Fig. 5h). To further make sure that the binding of C/EBPβ to SESN2 promoter is functional, the luciferase reporter plasmids containing wild-type (WT) or mutant-type (MUT) promoter of SESN2 were constructed to perform a dual-luciferase assay. As expected, the luciferase activity of the WT, but not the MUT plasmid, was significantly enhanced by rotenone treatment and suppressed after C/EBPβ knockdown (Fig. 5i). In brief, the above results indicated that the PERK-C/EBPβ-signaling pathway mediated SESN2 induction during rotenone or denervation-associated ERS.

### SESN2 attenuated ERS through AMPK/mTORC1 pathway

**Fig. 6:**
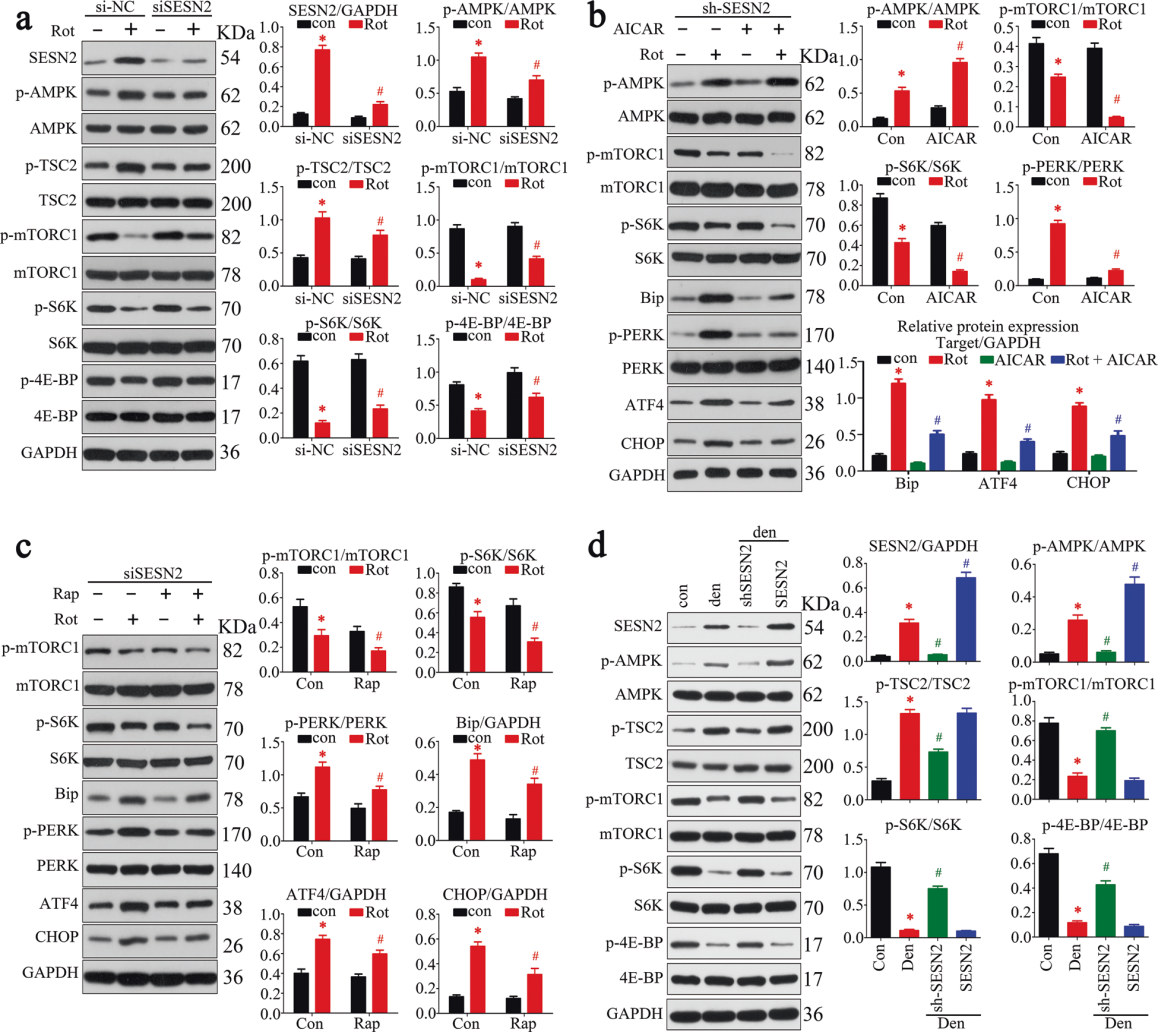
SESN2 attenuated ERS through AMPK/mTORC1 pathway. **a** The regulatory effect of SESN2 on the activity of AMPK/mTORC1 pathway was analyzed by western blot. Protein biosynthesis signaling downstream of mTORC1 was monitored by phosphorylation of p70 ribosomal S6K and 4E-BP. **b, c** After knockdown of SESN2, C2C12 cells were treated with AICAR (an AMPK activator) and rapamycin (an allosteric inhibitor of mTORC1) to analyze the protective role of AMPK–mTORC1 pathway against rotenone-induced ERS. **d** The regulatory effect of SESN2 on AMPK/mTORC1 pathway was further validated in denervated GAS. Data were presented as mean ± SD. *n* = 6. *P < 0.05 vs control. ^#^*P* < 0.05 vs rotenone treatment or denervation. Con control, Rot rotenone, Den denervation, Rap rapamycin.

To uncover the regulatory mechanism whereby SESN2 protected ER homeostasis, the AMPK/mTORC1 pathway, which had been well-documented to regulate protein biosynthesis, [20], was further explored. The results revealed that rotenone treatment activated AMPK and TSC2, inhibited mTORC1 phosphorylation. However, this pattern was nearly abolished in SESN2-knockdown cells. Consistently, phosphorylation of S6K and 4E-BP (downstream of AMPK/mTORC1) was blocked in rotenone-treated C2C12 cells and was partially reversed by SESN2 knockdown (Fig. 6a). To investigate whether the AMPK/mTORC1 pathway was involved in the protective effect of SESN2 against rotenone-induced ERS, we analyzed the levels of ERS marker proteins in SESN2-knockdown cells under AICAR (an AMPK activator) and rapamycin (an allosteric inhibitor of mTORC1) treatment. Our results suggested that compared with the rotenone-alone treatment group, cells treated with both rotenone and AICAR displayed lower phosphorylation levels of mTORC1 and S6K. Further, treatment with AICAR exerted consistently protective effect on ER homeostasis, evidenced by lower levels of Bip, PERK phosphorylation, ATF4, and CHOP (Fig. 6b). Similarly, rapamycin treatment also inhibited S6K phosphorylation and attenuated ERS (Fig. 6c). In agreement with the in vitro results, AMPK/mTORC1 pathway was also activated in denervated GAS and blocked after SESN2 knockdown (Fig. 6d). Taken together, these results demonstrated that the protective effect of SESN2 against denervation-induced ERS is dependent on the AMPK/mTORC1-signaling pathway.

### SESN2 promoted the elimination of dysfunctional mitochondria via mitophagy

**Fig. 7:**
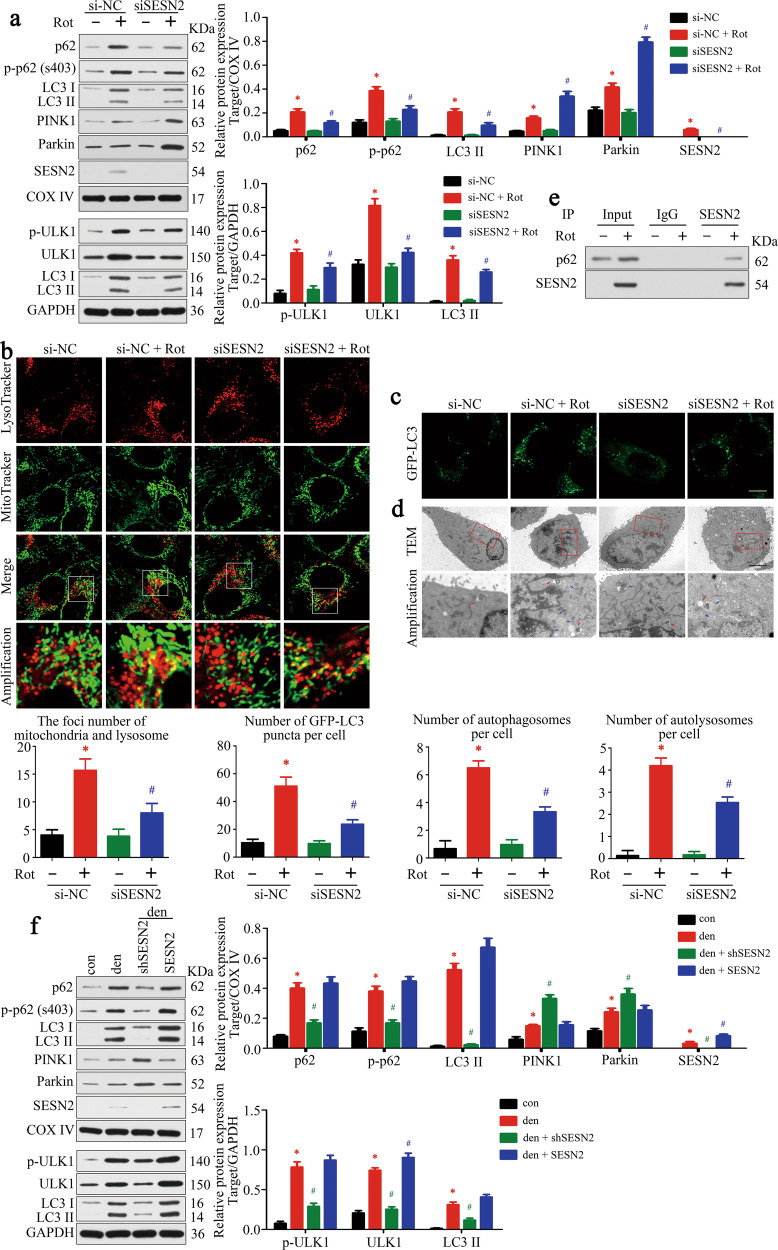
SESN2 promoted the eliminating of dysfunctional mitochondria via mitophagy. **a** Mitochondrial and total proteins in C2C12 cells were analyzed by western blot to explore the effect of SESN2 on mitophagy and autophagy signaling. **b** Costaining of mitochondria and lysosome by MitoTracke Green and LysoTracker Red. Part of the photographs (blue box) were amplified. The number of the overlaps (yellow dots) between the mitochondria and lysosome was counted to quantify the mitophagy activity. Scale bar 10 μm. **c** Representative images of C2C12 cells expressing GFP-LC3 under different treatments. The average number of GFP-LC3 puncta per cell was quantified. Scale bar 10 μm. **d** Autophagic activity in C2C12 cells was observed by TEM. Parts of the photos (red box) were amplified. Red and blue arrows indicated autophagosomes and autolysosomes, respectively. Numbers of autophagosomes and autolysosomes were statistically analyzed. **e** Immunoblot analysis of p62 in SESN2 immunoprecipitates (IP) in mitochondrial fraction and input. **f** The regulatory effect of SESN2 on mitophagy was further explored in denervated GAS. Data were presented as mean ± SD. *n* = 6. *P < 0.05 vs control. ^#^*P* < 0.05 vs rotenone treatment or denervation. Con control, Rot rotenone, Den denervation.

To investigate the exact mechanism whereby SESN2 protected mitochondrial function against denervation, mitophagy was focused considering its role in mitochondrial quality control. As expected, the mitochondria-located p62 and LC3 increased after denervation, and was reversed by SESN2 knockdown (Fig. 7a). Furthermore, mitochondria were widely tagged by lysosome after rotenone treatment, indicative of mitophagy activation. However, knockdown of SESN2 markedly reduced the overlap of mitochondria and lysosome, showing an inhibitory effect on mitophagy (Fig. 7b).

Next, we explored how mitophagy was regulated by SESN2. We first examined whether SESN2 was involved in autophagosome formation upon stimulation [21]. As former data had confirmed the regulatory effect of SESN2 on AMPK/mTORC1 pathway, we analyzed the expression of ULK1 and its phosphorylated version (Ser555). As expected, ULK1 and p-ULK1 were both elevated upon rotenone treatment and were reversed by SESN2 knockdown. LC3II, a quantitative index of autophagic activity, altered in a similar manner like ULK1 (Fig. 7a). The dynamic expression of LC3 after rotenone treatment with or without bafilomycin A1 was shown in Supplementary Fig. 4. Visualization of autophagic activity by GFP-LC3 was further performed and achieved consistent results with increased green fluorescence puncta upon rotenone treatment and suppressed by SESN2 knockdown (Fig. 7c). In addition, the ultrastructure of C2C12 cells was observed to validate the autophagic activity. As seen in Fig. 7d, abundant autophagosomes and autolysosomes were shown in rotenone-treated cells, mitophagosomes (autophagosomes enclosing mitochondria) could also be captured therein, whereas they were both reduced by SESN2 knockdown. Subsequently, we explored whether SESN2 was involved in mitochondrial priming during mitophagy. As shown in Fig. 7a, mitochondrial priming was initiated by PINK1 stabilization and the E3 ubiquitin ligase Parkin recruitment toward damaged mitochondria. Outer-membrane proteins of mitochondria were then ubiquitinated and induced translocation of the ubiquitin-binding receptor p62 to mitochondria membrane. Interestingly, this pattern was disturbed by SESN2 knockdown, in which PINK1/Parkin was elevated, while p62 was suppressed. Thus, we speculated that SESN2 might get involved in p62 translocation. Indeed, SESN2 was found to accumulate in mitochondria and to disappear after SESN2 knockdown, which was consistent with the variation of p62. Then mitochondrial fraction was subjected to Co-IP with SESN2 antibody, it was seen that the recruited SESN2 interacted with p62 in mitochondria, which was abolished in the absence of rotenone (Fig. 7e). In line with the reduced translocation of p62 caused by SESN2 knockdown, phosphorylation of p62 at specific sites (e.g., Ser403), which was reported to be required for the enclosure of mitochondria by autophagosomes, also decreased (Fig. 7a). Similar to the in vitro results, the effect of SESN2 on mitophagy was also validated in denervated GAS (Fig. 7f). Taken together, these findings suggested that SESN2 could promote mitophagy by facilitating autophagosome formation and the aggregation of p62 on mitochondria, thus reducing the accumulation of dysfunctional mitochondria.

## Discussion

Skeletal muscle atrophy induced by denervation is common for all kinds of muscles, though different types of fibers displayed distinct resistance to atrophy [22]. Mitochondria and ER are abundant in skeletal muscle and are necessary for its contractile activity and metabolism [23]. Previous researches had explored the vital roles of mitochondria and ER during muscle atrophy separately [6, 24]. However, their intrinsic association is usually ignored, which is improper considering their cooperation in calcium homeostasis, oxidative stress, lipid transport, and apoptosis [25]. A recent study indicated sestrin to act as a central regulator of anabolic and degradative signals that prevented atrophy in disused muscles. Sestrin prevents atrophy through mTORC1 inhibition and AKT activation, which upregulates autophagy and inhibits FoxO-regulated UPS separately. However, detailed mechanisms involved in sestrin induction and the protective effect on denervated muscle atrophy remain unclear [26]. In the present study, ERS and mitochondrial dysfunction were both validated in denervated muscle. SESN2, the stress-inducible protein, was found to be elevated by PERK-C/EBPβ axis upon ERS and relieved muscle atrophy by attenuating ERS, mitochondrial dysfunction, and the consequential apoptosis. Further, the mechanism underlying the protective role of SESN2 in ER homeostasis and mitochondrial quality was explored. On the one hand, SESN2 mediated the UPR through AMPK–mTORC1 pathway to reduce protein synthesis. On the other hand, SESN2 induced mitophagy by facilitating autophagosome formation and p62 aggregation to eliminate dysfunctional mitochondria. Altogether, the present study reveals an endogenous protection factor—SESN2 against denervation-induced muscle atrophy. Moreover, denervation-induced mitochondrial dysfunction and ERS may act as a potential target for the treatment or prevention of muscle atrophy.

According to existing evidence, SESN2 is always induced upon various stress, and exerts cytoprotective activity through regulation of protein synthesis, autophagy, and apoptosis [27]. Hence, we examined whether SESN2 is induced in denervated muscle and acts as an endogenous protective factor. To our surprise, SESN2, but not SESN1 and SESN3, was greatly elevated during the first two weeks after denervation, which was consistent with the period of muscle atrophy. Subsequent experiments further confirmed its protective role as knockdown of SESN2 significantly aggravated muscle atrophy. Here we noticed that overexpression of SESN2 just slightly reserved muscle atrophy (*P* > 0.05). Combined with our later results, we concluded that the endogenous increase of SESN2 might reach a saturated state and further addition of SESN2 exerted no further improvement on ERS, mitochondrial dysfunction, and apoptosis. Besides, a higher level of SESN2 induced the increase of autophagic activity, which added to protein degradation and might be detrimental to muscle weight.

As a serine–threonine protein kinase, mTOR was initially discovered in yeast mutants that was able to reverse the growth–inhibitory effect of rapamycin and then cloned in mammalian cells [20]. mTOR is usually assembled into two multiprotein complexes—mTORC1 (sensitive to rapamycin) and mTORC2 (insensitive to rapamycin). Activation of mTORC1 promotes protein synthesis via phosphorylation of the downstream proteins S6K and 4E-BP. AMPK, the regulator of cellular energy homeostasis, acts as the key attenuator of mTOR in cells. Previous research indicated that SESN2 suppressed protein synthesis via activation of AMPK, phosphorylation of TSC2, and subsequently inhibition of mTOR [28]. Here in our study, elevation of p-AMPK, p-TSC2, and reduction of p-mTORC1 were observed in denervated GAS and rotenone-treated C2C12 cells, which was followed by decreased phosphorylation of S6K and 4E-BP. Knockdown of SESN2 relieved the inhibition of mTORC1 and finally aggravated ERS. Pharmacological regulation of AMPK/mTORC1 pathway by AICAR and rapamycin in SESN2-knockdown cells mimicked the role of SESN2 and similarly attenuated ERS, further confirming the role of AMPK/mTORC1 pathway in SESN2-mediated ER homeostasis maintenance.

Mitochondrial dysfunction is characterized by the accumulation of mtROS, which leads to extensive oxidative damage of DNA, proteins, and lipids in the whole cell, including mitochondria [29]. SESN2 has been well-documented to protect cells from oxidative stress via suppressing intracellular ROS levels, though obvious lack of intrinsic catalytic antioxidant activity. A well-known pathway that mediates the antioxidant effect of SESN2 is Keap1/Nrf2 signaling. Generally, SESN2 triggers the autophagic degradation of Keap1 in a p62-dependent way and thus activates Nrf2, which subsequently upregulates the expression of various antioxidant genes through binding to AREs (antioxidant-response elements) [30, 31]. Here, in our present study, a different pathway that mainly acts through mitophagy is advanced to interpret the SESN2-induced ROS decrease. Once induced, SESN2 was transferred to the mitochondria and promoted the ubiquitin-mediated recruitment of p62. Further, interaction of SESN2 and p62 was confirmed and promoted the phosphorylation of p62 at Ser403, which was required for the enclosure of mitochondria by autophagosomes. Based on these results, we tested whether SESN2 works as a scaffold to enhance the originally weak binding of p62 to mitochondrial ubiquitinated proteins and thus activates mitophagy. Details of the interaction between SESN2, p62, and ubiquitinated proteins have not yet been explored due to limited time and materials. Further research on this issue will be carried out soon.

Significantly, this study had not explored the calcium dynamics, which has been well established to play an essential role in the coupling of muscle excitation and contraction [32]. Given the accumulating evidence suggesting the coordination of calcium dynamics through membrane-contact sites between ER and mitochondria (known as mitochondria-associated ER membranes) [33], it will be interesting to investigate the involvement of SESN2 in calcium homeostasis during denervated muscle atrophy in our future study.

In summary, this study revealed an endogenous protection mechanism of skeletal muscle against denervation-induced atrophy, where SESN2 mediated skeletal muscle adaptation to ERS and mitochondrial dysfunction through UPR and mitophagy.

## Materials and methods

### Animal procedures

Male C57BL/6 J mice of ten weeks, housed in a day–night cycle of 24 h, were purchased from SPF (Beijing) biotechnology co., LTD. Denervation was surgically conducted on the right hind legs of mice as priorly interpreted [34]. The mice were then randomly divided into four groups (6 mice/group): sham-operation group (control), denervation group, denervation + AAV-shSESN2 (adeno-associated virus, U6-MCS-CAG-EGFP; Genochem, China) group, and denervation + AAV-SESN2 group (CMV-betaGlobin-MCS-SV40 PolyA, Genochem). The sequence of shSESN2 primers was 5′-GCGTCTTTGGCATCAGATACG-3′. AAV9 injection of gastrocnemius was conducted three weeks ahead of schedule in order to construct SESN2-knockdown or overexpression models. In particular, 10 µl of virus (1.0 × 10^12^ vg/ml) were injected into each point around the gastrocnemius. To prevent the backflow of viral particles, the syringe was left in place for an extra five minutes after injection. In total, 4–6 injections were conducted on every limb. As for preparation of PERK and C/EBPβ-knockdown models, cholesterol-conjugated siRNA (10 nmol) and its negative control in 0.1 mL of saline buffer was injected into the gastrocnemius once every three days after denervation as reported previously [35]. The sequences of siRNAs were shown in Supplementary Table 2. Mice were euthanized at the indicated time, GAS, EDL, and SOL were removed, weighed, and frozen for the next experiments. The effects of gene transfection were confirmed by western blot or fluorescence (Supplementary Fig. 5).

The entire experimental process was executed in line with the guidelines of the Chinese National Institutes of Health. The approval of the experimental protocols was made through the Ethical Committee on Animal Experiments (Huazhong University of Science and Technology).

### Muscle mass measurements, H&E staining, ROS detection, and fiber-diameter quantification

Muscles (GAS, EDL, and SOL) of the denervation and control sides were harvested and weighted at the indicated time points. The wet-weight ratio (muscle weight of the denervation side divided by that of the control side) was adopted to evaluate muscle atrophy.

For H&E staining, muscles were fixed with paraformaldehyde (4%) for 24 h, and then dehydrated and embedded in paraffin. About 4-μm-thick cross-cutting slices of muscles were obtained and followed by H&E staining (Bioyear, China) according to the instructions.

Total levels of GAS ROS were measured via dihydroethidium (DHE; Beyotime, China). GAS was snap-frozen at liquid nitrogen immediately after being excised from the mice. Additionally, 10-μm-thick cross sections were prepared and incubated with 10 μM DHE at 37 °C for half an hour in the dark. Sections were then imaged using a fluorescence microscope.

For fiber-diameter quantification, muscle cross sections were stained for WGA to visualize fibers. ImageJ software was employed to qualify the minimal Feret’s diameter of fibers randomly selected in each group.

### Western blot and co-immunoprecipitation (Co-IP)

The following primary antibodies were used: MHC (R&D Systems, USA), SESN1 (Abcam), SESN2 (Abcam), SESN3 (Abcam), Bip (Abcam), CHOP (Abcam), TOM20 (Abcam), TIM23 (Abcam), Bax (Abcam), Bcl-2 (Abcam), cleaved-caspase 9 (Cell Signaling Technology, USA), cleaved-caspase 3 (Cell Signaling Technology), cytochrome c (Abcam), COX IV (Proteintech, China), PERK (Cell Signaling Technology), phospho-PERK (Cell Signaling Technology), C/EBPβ (Abcam), IRE1α (Cell Signaling Technology), ATF6 (Abcam), ATF4 (Abcam), AMPK (Cell Signaling Technology), phospho-AMPK (Cell Signaling Technology), TSC2 (Cell Signaling Technology), phospho-TSC2 (Cell Signaling Technology), mTORC1 (Cell Signaling Technology), phospho-mTORC1 (Cell Signaling Technology), S6K (Cell Signaling Technology), phospho-S6K (Cell Signaling Technology), 4E-BP (Cell Signaling Technology), phospho-4E-BP (Cell Signaling Technology), p62 (Cell Signaling Technology), p-p62 (Cell Signaling Technology), LC3B (Abcam), PINK1 (Abcam), Parkin (Abcam), ULK1 (Cell Signaling Technology), phospho-ULK1 (Cell Signaling Technology), and GAPDH (Proteintech). Primary antibody dilution factors were 1:3000 (all R&D Systems antibodies), 1:5000 (all Proteintech antibodies), or 1:1000 (all Abcam and Cell Signaling Technology antibodies).

Briefly, the protein was extracted with RIPA lysis buffer supplemented with 1% protease inhibitor (Roche, New Jersey). Equal amounts of protein (10–50 μg) were separated in a 10% SDS–polyacrylamide gel, and then transferred to nitrocellulose membranes (Merck Millipore, USA). The membranes were blocked in 5% w/v bovine serum albumin before incubation with the primary antibodies at 4 °C overnight. Then, the membranes were incubated with anti-rabbit or anti-mouse IgG-conjugated secondary antibodies (Abcam) for 1 h at room temperature and visualized using the Immobilon ECL substrate kit (Merck Millipore). For quantitative analysis of mitochondrial proteins, Mitochondria Isolation Kits (Solarbio, China) were employed to extract mitochondria before protein quantification.

For Co-IP analysis, cells were lyzed by Triton-lysis buffer and then centrifuged (12,000 × *g*) at 4 °C for 10 min. Protein A/G PLUS-Agarose (Santa Cruz, USA) was then added to the supernatant and incubated with antibodies specific for p62 and SESN2 for 24 h. The immunoprecipitants were released and analyzed by Western blot.

### Transmission electron microscope (TEM) observation

The morphology of ER and mitochondria in GAS was visualized by TEM. Briefly, muscles were cut into 1.0-mm^3^ cubes, fixed with 2% glutaraldehyde at 4 °C overnight, and then postfixed in 1% OsO_4_ for 2 h. After dehydration and embedding in epoxy resin, ultrathin sections (60 nm) were prepared and stained with uranyl acetate and lead citrate. Images were captured under a transmission electron microscope (HT-7700, Hitachi, Japan). Mitochondria number was quantified in a blind way described before [36]. Similar procedures were performed for the observation of C2C12 cells.

### Measurement of Δψm

Δψm in C2C12 cells and GAS was evaluated by the JC-1 probe (MedChemExpress, USA). Briefly, C2C12 cells were incubated with JC-1 for 20 min at 37°C and then washed three times with PBS before imaging. Confocal laser scanning microscopy was used for fluorescent image capture and intensity analysis. Red fluorescence and green fluorescence represent the polymer and monomer forms of JC-1, respectively. The ratio of red-to-green fluorescence intensity was calculated to describe Δψm levels. At least 30 cells in each group were selected randomly to generate the final results.

### Cell culture and transfection

C2C12 cells were obtained from iCell Bioscience Inc. and maintained with Dulbecco’s modified Eagle’s medium (Gibco, USA) containing 10% fetal bovine serum (Gibco) and 100 units/mL penicillin/100 μg/mL streptomycin solution (Sangon Biotech, China) with 5% CO_2_ in a 37 °C cell culture incubator.

For gene knockdown, short-interference RNAs for mouse SESN2 (siSESN2), PERK (siPERK), IRE1α (siIRE1α), ATF6 (siATF6), C/EBPβ (siC/EBPβ), ATF4 (siATF4), CHOP (siCHOP), and the corresponding negative controls (si-NC) were synthetized by RiboBio Co., LTD (Guangzhou, China). Cell transfection was conducted according to the instruction of Lipofectamine 2000 (Invitrogen) with a final siRNA concentration of 50 nM. Cells were harvested at 48 h after transfection for the following experiments. The gene-silencing effect was validated by western blot (Supplementary Fig. 6). siRNAs with maximum inhibitory effect were adopted for subsequent research. The sequences of siRNAs were shown in Supplementary Table 2.

### Detection of mitochondrial ROS level in C2C12 cells

Detection of mtROS was performed using the fluorogenic dye MitoSOX™ Red (Thermo Fisher, USA), which was specifically targeted to mitochondria in live cells. Briefly, 5 mM stock solution and 5 μM working solution of MitoSOX™ were prepared according to the manufacturer’s instruction. Cells were incubated with working solution for 10 min in the dark at 37 °C and then washed three times with a warm buffer. Live-cell images were captured by a confocal laser scanning microscope. At least 30 cells in each group were selected randomly to generate the final results.

### ChIP assay

ChIP was conducted using the Magna ChIP kit (Millipore). Briefly, the nucleic acids and proteins in cells were cross-linked by formaldehyde incubation and then quenched by glycine buffer (10×). Sonication was employed to create DNA fragments (200-400 bp). The anti-C/EBPβ, anti-RNA polymerase II (Pol II) and anti-acetylated histone H3 (Lys9, Cell Signaling Technology) antibodies were then adopted to precipitate the DNA fragments. Anti-IgG (Abcam) antibodies were applied as controls. Finally, qRT-PCR (Forward primer: 5′-GACTCCTTGGGCTGTCACTC-3′; Reverse primer: 5′-CCAATCAGCATCGACAAGCG-3′) analysis was used for determination of precipitated DNAs.

### Luciferase reporter assay

SESN2 promoter region (2.0-kb sequence upstream of transcription-initiation site) that contains putative binding site (wild type) or mutant site (mutant type) was constructed into pGL3-based luciferase reporter vector (namely pGL3-SESN2). C2C12 cells were co-transfected with pGL3–SESN2 and siC/EBPβ. After the cells had been cultured for 48 h, Renilla and firefly luciferase activities were measured by the Dual-Luciferase Reporter Assay System Kit (Promega, USA). All experiments were repeated three times independently.

### Mitochondria and lysosome imaging

Specific probes—MitoTracker Green and LysoTracker Deep Red (Invitrogen, USA) were utilized to visualize mitochondria and lysosomes, separately. Cells were incubated in the dark at 37 °C by freshly prepared MitoTracker Green or LysoTracker Deep Red working solution with concentrations of 1 μM and 0.5 μM, respectively. After 20 min, cells were washed three times and then captured by a confocal laser scanning microscope. At least 30 cells in each group were selected randomly to generate the final results. Yellow dots represented the colocalization of mitochondria and lysosomes.

### Autophagic flux analysis

Autophagic flux in C2C12 cells was detected by using the GFP-LC3 adenovirus (Hanbio, China). After placing in a 24-well plate at a density of 1 × 10^4^ cells/dish, the cells were incubated with GFP-LC3 adenovirus for 24 h and then used for subsequent experiments. Autophagic flux was observed under a confocal laser scanning microscope. The green puncta indicated autophagosomes.

### Statistical analysis

Statistical analysis of three independent experiments with at least three technical repetitions was conducted using GraphPad Prism 7.0 (GraphPad, USA), and the statistical data were expressed as mean ± standard deviation. Differences were evaluated by one-way analysis of variance followed by Tukey’s test. *P* value less than 0.05 was considered as statistically significant.

## Supplementary information

Supplementary Materials

## Data Availability

All data generated or analyzed during this study are included in this published article [and its supplementary information files].
