# Supplementary material for: SESN2 protects against denervated muscle atrophy through unfolded protein response and mitophagy
Source: Cell Death Dis. 2021 Aug 24;12(9):805. doi: 10.1038/s41419-021-04094-9 (PMC8384848; doi:10.1038/s41419-021-04094-9)
Supplement: Supplementary file 1 — Supplementary Materials [file 41419_2021_4094_MOESM1_ESM.docx]

Supplementary Materials for

**SESN2 protects against** **denervated muscle atrophy through unfolded protein response and mitophagy**

Xiaofan Yang^1^ · Pingping Xue^2^ · Meng Yuan^1^ · Xiang Xu^1^ · Cheng Wang^1^ ·

Wenqing Li^3^ · Hans-Günther Machens^4^ · Zhenbing Chen^1,^*

^1^Department of Hand Surgery, Union Hospital, Tongji Medical College, Huazhong University of Science and Technology, Wuhan 430022, China

^2^Department of Pharmacy, Tongji Hospital, Tongji Medical College, Huazhong University of Science and Technology, Wuhan 430030, China

^3^Department of Hand and Foot Surgery, Huazhong University of Science and Technology Union Shenzhen Hospital, Shenzhen, Guangdong, China

^4^Department of Plastic and Hand Surgery, Technical University of Munich, Munich 81675, Germany

*Corresponding author: Zhenbing Chen ([zbchen@hust.edu.cn](mailto:zbchen@hust.edu.cn))

**This file includes:**

Supplementary Materials and Methods

Supplementary Figures 1 to 6

Supplementary Tables 1 to 2

**Supplementary Materials and Methods**

Immunofluorescence

Paraffin-embedded GAS underwent cross-sectional cuts, which were made into 4-μm sections as previously stated. Following antigen retrieval, permeabilization, and goat serum blocking, primary antibody (rabbit anti-WGA, 1:100; Abcam, UK) incubation was implemented at 4°C. Next, samples were stained for one hour based on secondary Alexa Fluor 488-conjugated secondary antibodies (1:300, Invitrogen, USA), followed by 5 minutes DAPI/PI (Sigma, USA) staining. The samples were then imaged with a fluorescence microscope, and similar processes were oriented with C2C12 cells.

Quantitative reverse transcriptase/real-time PCR (qRT–PCR)

Total RNA was extracted by TRIzol reagent (Invitrogen, USA). One microgram of RNA was reverse transcribed into cDNA using a first strand cDNA synthesis kit (Toyobo, Japan) according to the manufacturer’s instructions. RNA extraction and reverse-transcription were performed by PrimeScriptTM RT reagent Kit (Takara, Japan). qRT-PCR was performed on an ABI StepOne Plus using SYBR Green® Premix Ex Taq (Takara). Expression levels were normalized against β-actin. The set of deltaCq replicates (Cq values for each sample normalized against the geometric means of the reference genes) for control and tested samples were used for statistical testing and estimation of the p values. Shown are fold changes versus untreated controls. The primers were purchased from Tsingke Biological Technology (Beijing, China), and the sequences are detailed in Supplementary Table 1.

ATP level

ATP levels in mice GAS were measured by ATP assay kit (Beyotime). Briefly, The GAS tissues were homogenized in ATP assay lysis buffer and the supernatant was collected by centrifuging at 12,000×g for 5 min at 4 °C and quantified using BCA assay. Then, 100 μl ATP detection reagent was added to 100 μl supernatant, and the firefly luciferase activity was detected and analyzed by luminescence spectrometry (EnSpire, USA). The ATP level was normalized to cellular protein concentration and expressed as percentage relative to the control. Similar procedures were performed in C2C12 cells.

Cell death ELISA

Gastrocnemius were extracted, blotted to remove excess liquid, minced, and homogenized. Differential centrifugation was then used to isolate cytosolic extracts and protease inhibitors were added to prevent protein degradation. A cell death detection ELISA (Roche, USA) was used to quantitatively determine the apoptotic DNA fragmentation by measuring the cytosolic histone-associated mono- and oligonucleosomes. The assay was performed according to the manufacturer’s instructions. Final absorbance was determined with a microplate reader at 405 nm. Data were normalized to total protein concentration by BCA assay. A wavelength of 490 nm was used as the reference wavelength.

Apoptosis evaluation by TUNEL staining and flow cytometry

Apoptosis was measured by TUNEL staining and flow cytometry (FCM). TUNEL staining was detected by TdT enzyme according to the manufacturer’s instructions, and TUNEL-positive percentage was calculated per section. Flow cytometry was performed by labelling Annexin V and propidine iodide (PI) according to manufacturer’s instructions (BD Biosciences, USA). Data were analysed by FlowJo software. Cells were considered apoptotic if they were annexin V-positive/PI-negative, while double-positive cells reflected necrosis or late apoptosis.

**Supplementary Figures**


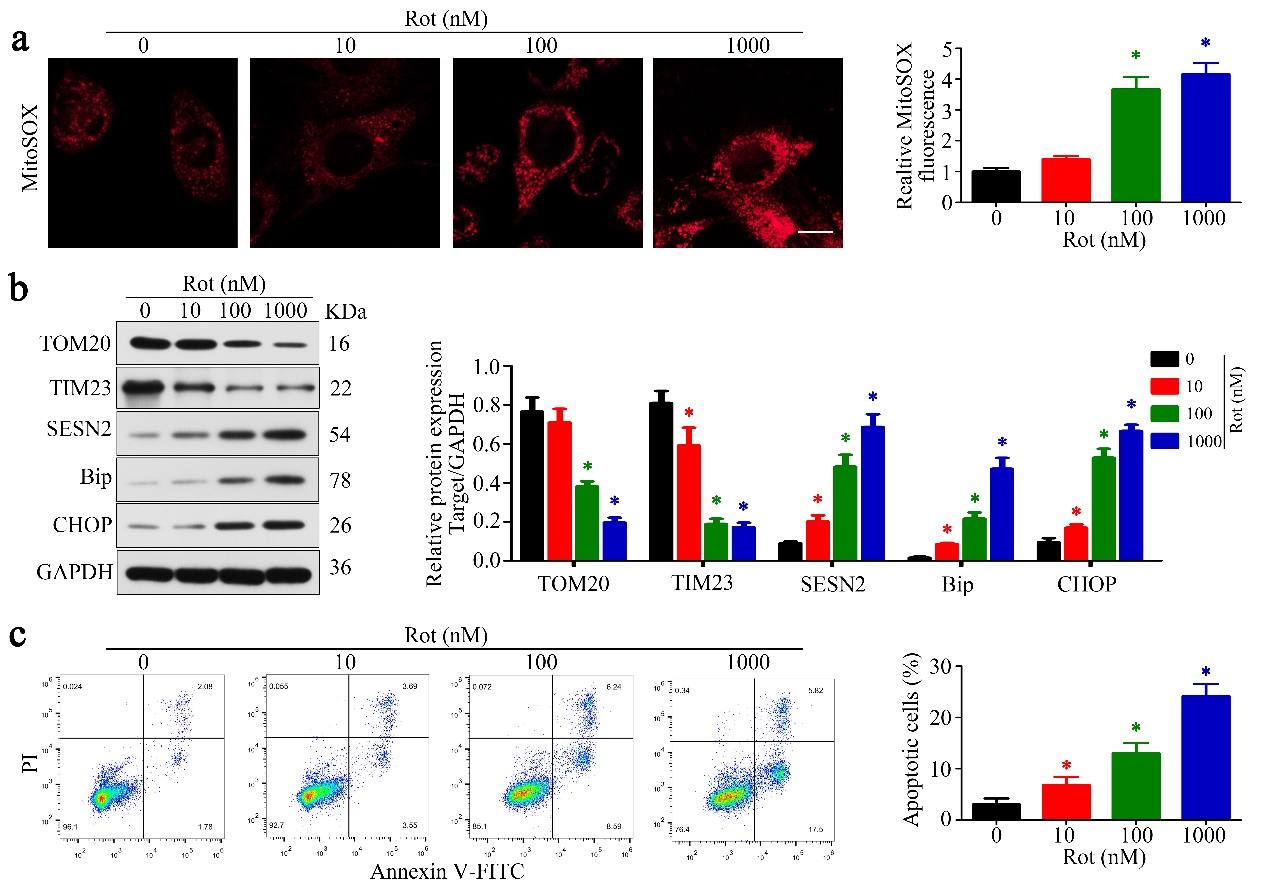


**Fig. 1** ERS, mitochondrial dysfunction and apoptosis was activated in rotenone-treated C2C12 cells. **a** Mitochondrial ROS levels in C2C12 cells were detected by MitoSOX Red labelling. Relative MitoSOX fluorescence was analyzed. Scale bar 10 μm. **b** Western blot analysis confirmed the elevation of SESN2 in rotenone-treated C2C12 cells. ER and mitochondria homeostasis were also investigated by Bip, CHOP, TOM20 and TIM23 separately. **c** FCM analysis of the apoptosis of C2C12 cells. The apoptotic rate was analyzed. Data were presented as mean ± SD. *P < 0.05 vs control (0 nM rotenone treatment). Rot, rotenone.


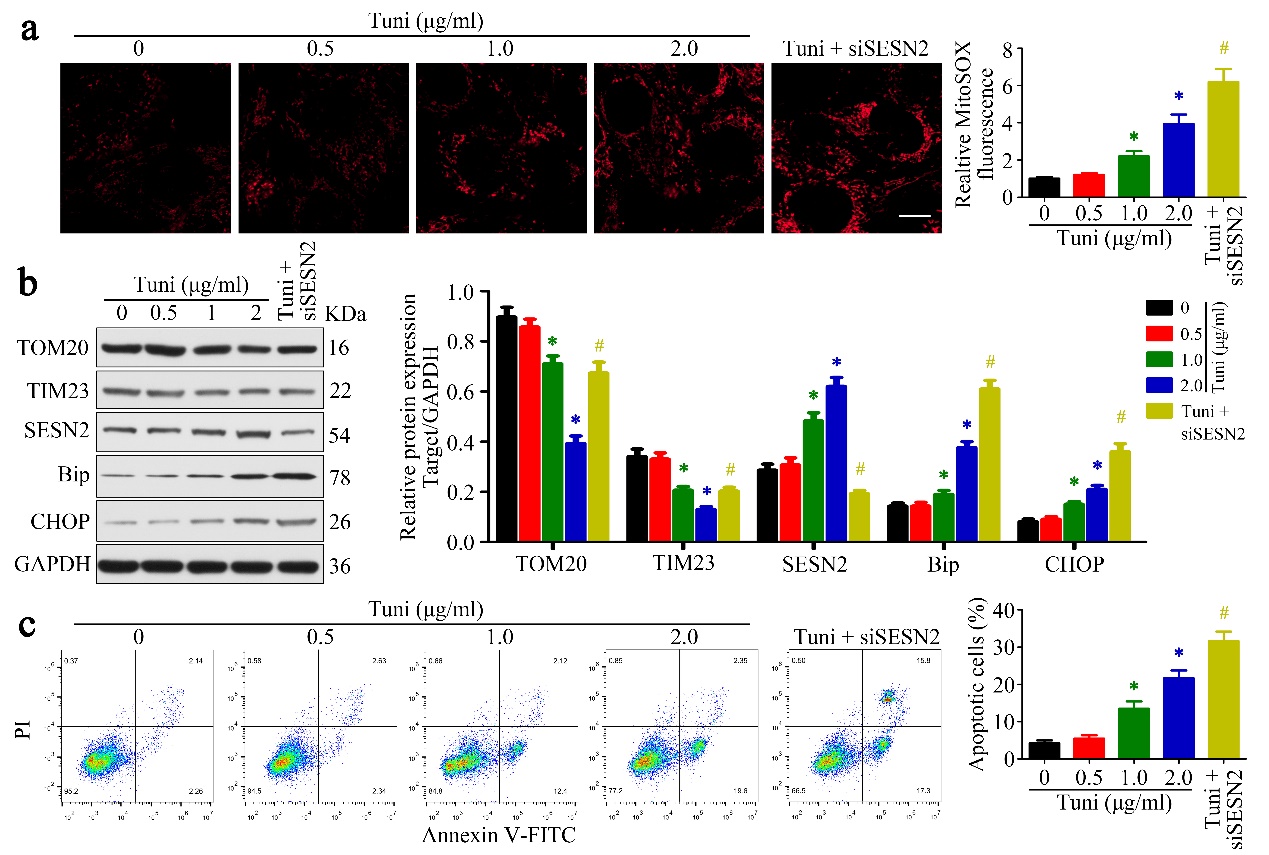


**Fig. 2** ERS, mitochondrial dysfunction and apoptosis was activated in tunicamycin-treated C2C12 cells. **a** Cells were incubated with a concentration gradient of tunicamycin for 24 h. Knockdown of SESN2 by siRNA was transfection was conducted 48 h before tunicamycin treatment. Mitochondrial ROS levels in C2C12 cells were detected by MitoSOX Red labelling. Relative MitoSOX fluorescence was analyzed. Scale bar 10 μm. **b** Western blot analysis confirmed the elevation of SESN2 in rotenone-treated C2C12 cells. ER and mitochondria homeostasis were also investigated by Bip, CHOP, TOM20 and TIM23 separately. **c** FCM analysis of the apoptosis of C2C12 cells. The apoptotic rate was analyzed. Data were presented as mean ± SD. *P < 0.05 vs control (0 nM tunicamycin treatment). ^#^P < 0.05 vs tunicamycin treatment group (2 μg/ml). Tuni, tunicamycin.


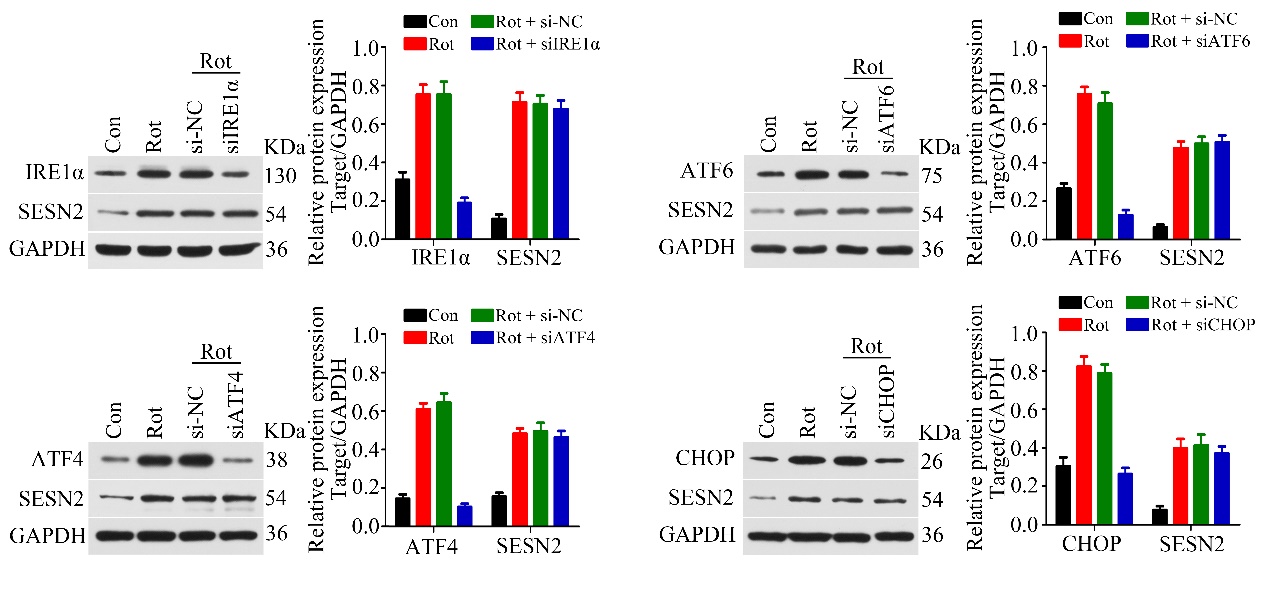


**Fig. 3** Knockdown of the ERS sensors IRE1, ATF6 and the transcription factors (ATF4, CHOP) downstream of PERK were achieved and their regulatory effect on SESN2 expression was confirmed by western blot.


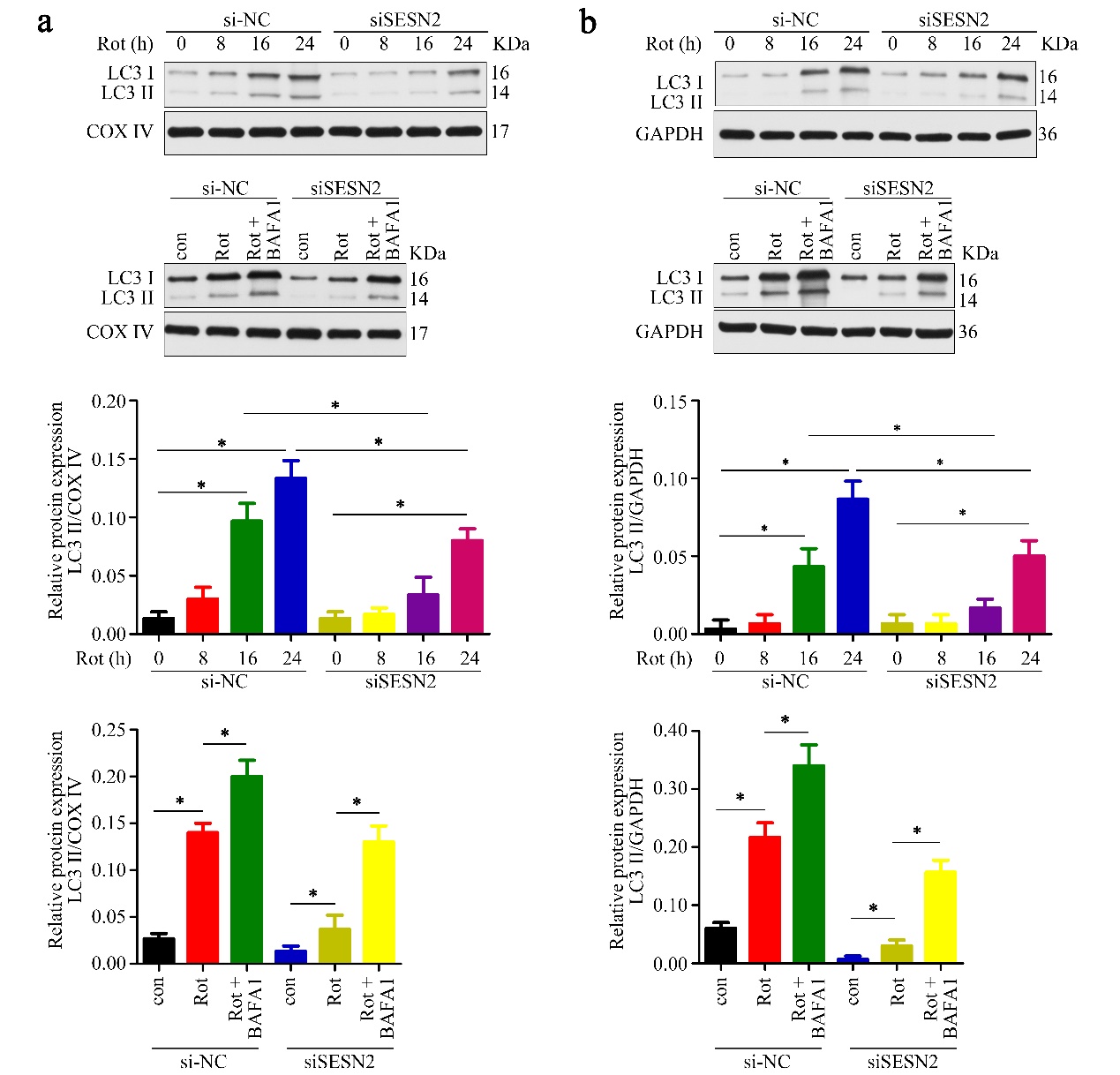


**Fig. 4** Dynamic expression of LC3 in C2C12 cells was detected by western blot. **a** Expression of LC3 in mitochondrial subfraction. **b** Expression of LC3 in total proteins. LC3-II levels increased over time and were further enhanced by bafilomycin A1. Data were presented as mean ± SD. *P < 0.05. Con, control; Rot, rotenone; BAFA1, bafilomycin A1.


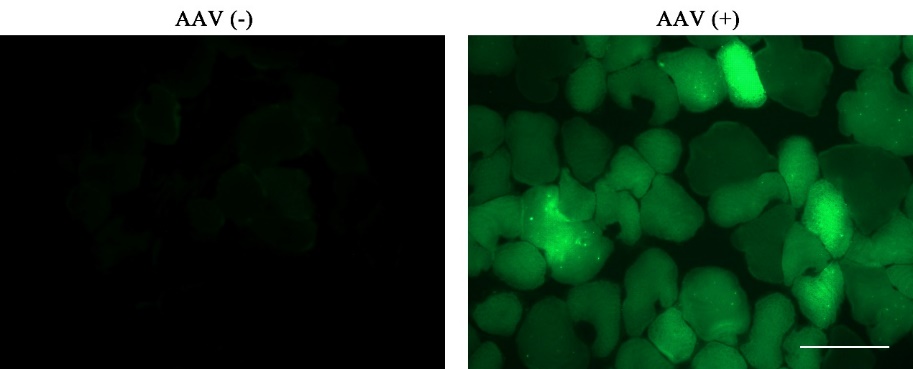


**Fig. 5** The effect of AAV-shSESN2 (U6-MCS-CAG-EGFP) transfection in GAS was validated by green fluorescence. Scale bar 25 μm.


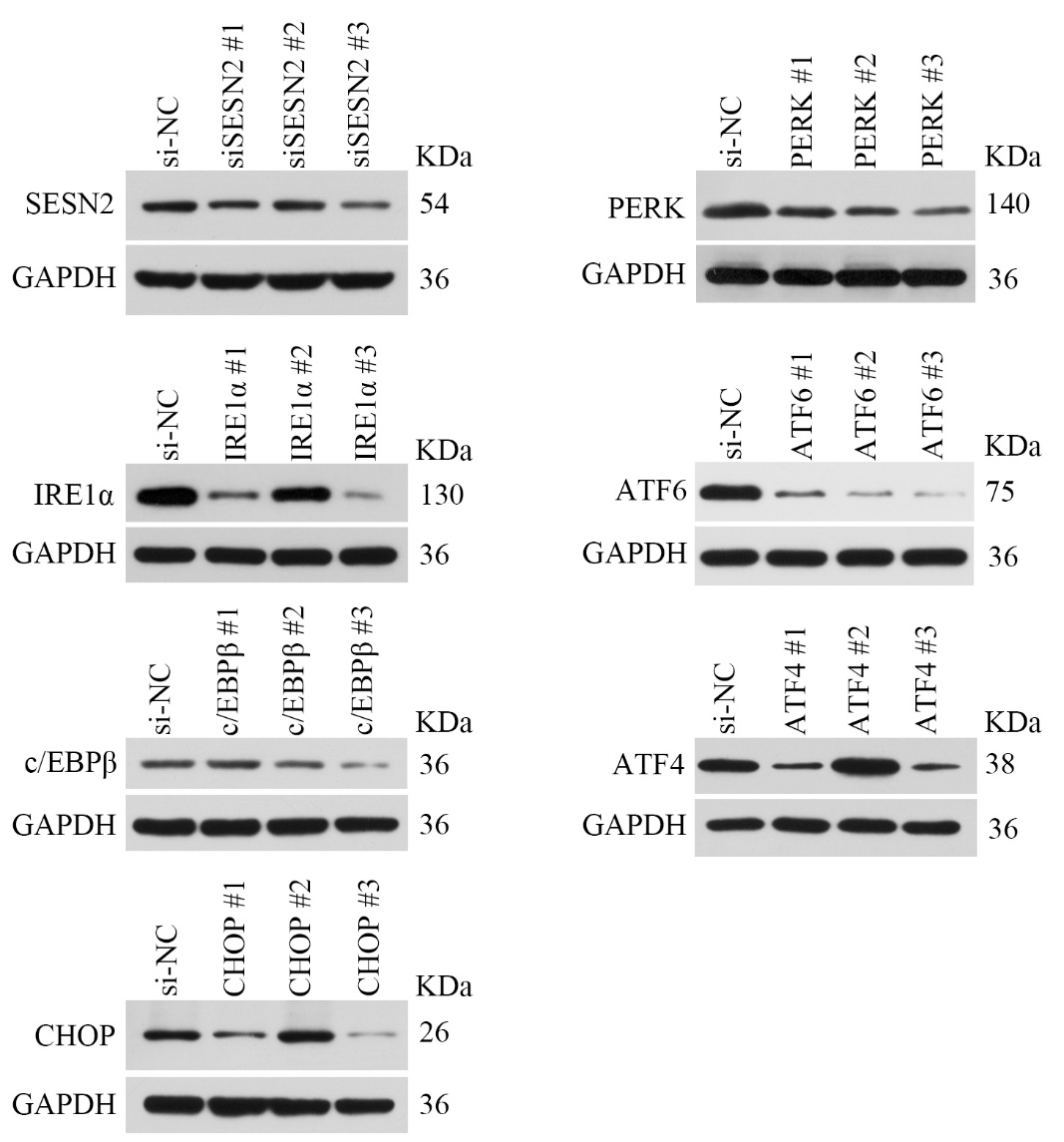


**Fig. 6** The inhibitory effect of siRNAs in C2C12 was validated by western blot.

**Supplementary Tables**

**Table 1. The sequences of PCR primers.**

| **Primers** | **Sequences** |
| --- | --- |
| SESN1 | Forward: 5′-GGCCAGGACGAGGAACTTG-3′ |
|  | Reverse: 5′-AAGGAGTCTGCAAATAACGCAT-3′ |
| SESN2 | Forward: 5′-TCCGAGTGCCATTCCGAGAT-3′ |
|  | Reverse: 5′-TCCGGGTGTAGACCCATCAC-3′ |
| SESN3 | Forward: 5′-CGGAAGGACAAAAGAATCCGA-3′ |
|  | Reverse: 5′-GTTCATCCGCCGTATTTGCT-3′ |
| β-actin | Forward: 5’-GGCTGTATTCCCCTCCATCG-3’ |
|  | Reverse: 5’-CCAGTTGGTAACAATGCCATGT-3’ |

**Table 2. The sequences of siRNAs.**

| **SiRNA Targets** | **Sequences** |
| --- | --- |
| SESN2-siRNA#1 sense | 5′-GGAAGAGATCCTTCGGGAA-3′ |
| SESN2-siRNA#2 sense | 5′-GCGTCTTTGGCATCAGATA-3′ |
| SESN2-siRNA#3 sense | 5′-CCACTCAGAGAAGGTTCAT-3′ |
| PERK -siRNA#1 sense | 5′-GCAGGTCCTTGGTAATCAT-3′ |
| PERK-siRNA#2 sense | 5′-CCTTTAAACCGGGTGGAAA-3′ |
| PERK-siRNA#3 sense | 5′-GCCATACTACAAGAGAGAA-3′ |
| IRE1α-siRNA#1 sense | 5′-CCATCAACTTCCCTTCTAT-3′ |
| IRE1α-siRNA#2 sense | 5′-GCTAACGCCTACTCTGTAT-3′ |
| IRE1α-siRNA#3 sense | 5′-GCGTCACCATTGGAGACAA-3′ |
| ATF6-siRNA#1 sense | 5′-GCAAAGCAGCAGTCGATTA-3′ |
| ATF6-siRNA#2 sense | 5′-GCCACCAGAAGTATGGGTT-3′ |
| ATF6-siRNA#3 sense | 5′-GCTGTCCAGTACACAGAAA-3′ |
| c/EBPβ-siRNA#1 sense | 5′-CCATGGAAGTGGCCAACTT-3′ |
| c/EBPβ-siRNA#2 sense | 5′-ACAAGGCCAAGATGCGCAA-3′ |
| c/EBPβ-siRNA#3 sense | 5′-CCTGCGGAACTTGTTCAAG-3′ |
| ATF4-siRNA#1 sense | 5′-GCTGCTTACATTACTCTAA-3′ |
| ATF4-siRNA#2 sense | 5′-CCACTCCAGAGCATTCCTT-3′ |
| ATF4-siRNA#3 sense | 5′-GGCCAAGGAGATCCAGTAT-3′ |
| CHOP-siRNA#1 sense | 5′-GCTCTCCAGATTCCAGTCA-3′ |
| CHOP-siRNA#2 sense | 5′-GCTAGCTGAAGAGAACGAG-3′ |
| CHOP-siRNA#3 sense | 5′-TCAGAGTTCTATGGCCCAG-3′ |
| NC-siRNA sense | 5′-UUCUCCGAACGUGUCACGUUU-3′ |
